# Draft genome sequences of three *Lactobacillus crispatus* strains, isolated from the female urinary tract

**DOI:** 10.1128/mra.00334-24

**Published:** 2024-05-29

**Authors:** Cerena Sedano, Natalie Stegman, Maria Steiling, Briana Jackson, Catherine Putonti

**Affiliations:** 1 Department of Biology, Loyola University Chicago, Chicago, Illinois, USA; 2 Bioinformatics, Biology, Loyola University Chicago, Chicago, Illinois, USA; Department of Biological Sciences, Wellesley College, Wellesley, Massachusetts, USA

**Keywords:** *Lactobacillus crispatus*, urinary tract, urobiome

## Abstract

*Lactobacillus crispatus is* a frequent member of the female urogenital microbiota. Here, we present the draft genome assemblies of three *L. crispatus* strains: UMB4356, UMB5661, and UMB6244. All strains were isolated from voided urine samples from females with type 2 diabetes.

## ANNOUNCEMENT

*Lactobacillus crispatus* is a frequent member of the female urogenital microbiota and is attributed to maintaining a “healthy” community (1, 2). When *L. crispatus* is exposed to infected human bladder epithelial cells *in vitro*, it significantly reduces the load of uropathogenic *Escherichia coli* (3). Premenopausal women given *L. crispatus* probiotic suppositories have a reduced incidence of recurrent urinary tract infections (4), and it also has been shown to prevent bacterial vaginosis and vulvovaginal atrophy (5, 6). These effects are likely the result of antimicrobials, for example, lactic acid, hydrogen peroxide, bacteriocins, and phenyl-lactic acid, produced by *L. crispatus* (7, 8). Here, we present the draft genome sequences of three *L. crispatus* strains isolated from voided urine samples from females with type 2 diabetes.

Strains UMB4356, UMB5661, and UMB6244 were isolated using the enhanced quantitative urine culture (EQUC) method (9), which isolates bacteria from urine samples using a variety of culture conditions and volumes of sample, as part of a prior study (Loyola University Chicago, IRB # 204197) (10). These isolates were identified as *L. crispatus via* MALDI-TOF, as previously described (11), and stored at −80°C in the Loyola Urinary Education and Research Collaborative (LUEREC) collection. The freezer stocks were obtained from this collection and streaked on deMan-Rogosa-Sharpe (MRS) agar plates and incubated under 5% CO_2_ at 35°C for 48 hours. Then, MRS medium supplemented with 1  mL/L of Tween 80 (Sigma-Aldrich) was inoculated with a single colony and incubated as described above. DNA was extracted using the DNeasy blood and tissue kit (Qiagen) following the manufacturer’s protocol for Gram-positive bacteria. DNA was quantified using a Qubit fluorometer.

DNA library preparation and sequencing were conducted by SeqCenter (Pittsburgh, PA USA). Libraries were prepared using the tagmentation-based and PCR-based Illumina DNA Prep kit (Ilumina, Inc., San Diego, CA USA) and custom IDT 10 bp unique dual indices (UDI) with a target insert size of 320 bp and sequenced on the Illumina NovaSeq 6000 platform, producing 2 × 151 bp paired-end reads. Raw reads were trimmed using BBDuk with default parameters (part of the BBMap suite, v39.01; sourceforge.net/projects/bbmap/), and assembled using SPAdes v3.15.5 with the --only-assembler option (12). Genome coverage was calculated using the BBWrap script in BBMap. Genome assemblies were annotated *via* the Prokaryotic Genome Annotation Pipeline (PGAP) v6.6 (13). Genome sequencing and assembly statistics are listed in Table 1.

**TABLE 1:** Genome sequencing and assembly statistics of the three urinary *L. crispatus* strains

Strain	# of Raw reads	# of Contigs	Length of genome (bp)	N50 (bp)	GC content	Coverage	ANI to *L. crispatus* VMC3	SRA accession	GenBank assembly accession
UMB4356	2,480,522	47	2,046,463	103,963	36.50%	175.871×	96.97%	SRR26087529	GCA_032376685.1
UMB5661	2,660,172	153	2,340,562	31,638	36.40%	161.031×	99.97%	SRR26087535	GCA_032376945.1
UMB6244	2,892,680	146	2,339,563	34,613	36.40%	175.193×	96.46%	SRR26087527	GCA_032376885.1

The average nucleotide identity (ANI) for each draft genome against the strain *L. crispatus* VMC3 (GCA_001541385.1) was calculated using JSpeciesWS (ANIb method) (14) (Table 1), and all were above the 95% species-threshold (15). Biosynthetic gene clusters were also identified in these genomes *via* antiSMASH v7 with default parameters (16). *L. crispatus* UMB4356 has two ribosomally synthesized and post-translationally modified peptides (RiPP)-like clusters, and UMB5661 and UMB6244 have three RiPP-like clusters (16). Overall, the sequencing of these samples brings further insight into the putative bactericidal effects of *L. crispatus* on uropathogens.

## Data Availability

Raw reads and draft assemblies have been deposited in GenBank. Table 1 lists the SRA accession numbers and genome assembly accession numbers.
